# Development of poly(methyl methacrylate)/poly(lactic acid) blend as sustainable biomaterial for dental applications

**DOI:** 10.1038/s41598-023-44150-2

**Published:** 2023-10-06

**Authors:** Taksid Charasseangpaisarn, Chairat Wiwatwarrapan, Pasutha Thunyakitpisal, Viritpon Srimaneepong

**Affiliations:** 1 Dental Biomaterials Science, Graduate School, Chulalongkorn University, Bangkok, 10330 Thailand; 2 College of Dental Medicine, Rangsit University, Pathum Thani, 12000 Thailand; 3 Chula Unisearch, Chulalongkorn University, Bangkok, 10330 Thailand; 4 Institute of Dentistry, Suranaree University of Technology, Nakhon Ratchasima, 30000 Thailand; 5 Department of Prosthodontics, Faculty of Dentistry, Chulalongkorn University, 34 Henri-Dunant Road, Wangmai, Patumwan, Bangkok, 10330 Thailand

**Keywords:** Dentistry, Dental materials, Dental biomaterials

## Abstract

Poly(lactic acid) (PLA) is gaining popularity in manufacturing due to environmental concerns. When comparing to poly(methyl methacrylate) (PMMA), PLA exhibits low melting and glass transition temperature (T_g_). To enhance the properties of these polymers, a PMMA/PLA blend has been introduced. This study aimed to investigate the optimal ratio of PMMA/PLA blends for potential dental applications based on their mechanical properties, physical properties, and biocompatibility. The PMMA/PLA blends were manufactured by melting and mixing using twin screw extruder and prepared into thermoplastic polymer beads. The specimens of neat PMMA (M100), three different ratios of PMMA/PLA blends (M75, M50, and M25), and neat PLA (M0) were fabricated with injection molding technique. The neat polymers and polymer blends were investigated in terms of flexural properties, T_g_, miscibility, residual monomer, water sorption, water solubility, degradation, and biocompatibility. The data was statistically analyzed. The results indicated that T_g_ of PMMA/PLA blends was increased with increasing PMMA content. PMMA/PLA blends were miscible in all composition ratios. The flexural properties of polymer blends were superior to those of neat PMMA and neat PLA. The biocompatibility was not different among different composition ratios. Additionally, the other parameters of PMMA/PLA blends were improved as the PMMA ratio decreased. Thus, the optimum ratio of PMMA/PLA blends have the potential to serve as novel sustainable biomaterial for extensive dental applications.

## Introduction

PMMA, also known as Poly(methyl methacrylate), has long been used extensively in dentistry including denture base, restorations. It is valued for its durability, strength, and biocompatibility which makes it a safe and effective material of choice for oral health care. Additionally, PMMA can also be easily colored to match the color of the adjacent teeth, producing a solution that is both natural-looking and aesthetically acceptable^1^. As a result, PMMA continues to be a preferred material in the dental industry. However, the residual monomer which leaching from PMMA still remains as health risk since it causes allergic reactions and toxic to human cells^2^. PMMA used in dentistry is available in various forms depending on the manufacturing processes (e.g., powder and liquid for compression molding or injection molding, polymer disc and liquid oligomer for digital manufacturing). The majority of these materials are semi-interpenetrating network (semi-IPN) so that the waste from manufacturing cannot be recycled^3^.

Recently, concern about environmental pollution has drastically increased, leading to high demand for sustainable polymeric materials. Sustainable polymers are polymers that are derived from natural resources or synthesis biopolymer. The major advantage of sustainable polymers is environment-friendly because they can be recycled, biodegraded, or bio-composted^4,5^. Poly(lactic acid) or PLA is one of the most popular bio-based and biodegradable polymers which has been used in both industrials and medical applications due to its good mechanical properties, and biocompatibility^6^.

PLA and its derivatives have been employed in dentistry as suturing materials, surgical membranes, scaffolds, orthodontic appliance, provisional restoration, and dental model^7–9^. PLA is widely used for materials in digital manufacturing, both subtractive and additive manufacturing. However, because of its low glass transition temperature (T_g_), which is claimed to be between 55 and 60°C^10^ and low toughness, PLA are limited to temporary or non-loaded applications. Moreover, PLA is susceptible to hydrolytic and enzymatic degradation^11,12^. According to Charasseangpaisarn et al., the flexural properties of 3D-printed PLA by fuse-deposition modeling (FDM) were lower than that of conventional heat polymerized PMMA. Furthermore, the flexural properties also decreased when evaluated in the high temperature environment^13^. Thus, the improvement of thermal properties, especially glass transition temperature, is necessary for using PLA as long term temporary or permanent restorative material.

An easy way to modify the properties of polymers is polymer blending. Many previous studies show the improved the T_g_ of PLA by mixing with other polymers, including PMMA^14–16^. The previous studies showed that blending PMMA to PLA could be either miscible or immiscible depending on many factors such as specific interaction, blending method^11,16–18^. The miscible PMMA/PLA blend can improve the T_g_, and tensile strength of PLA including having synergistic effect of polymer blend^14^. However, most studies on PMMA/PLA blend were investigated for industrial usage but not for dental use. Therefore, the hypothesis of this study was that optimal ratio of PMMA/PLA blend could improve both physical and mechanical properties of the polymer blend compared to the neat PMMA and PLA.

## Results

### Flexural properties

The results from Table 2 showed that the different melt temperatures had an influence on the flexural properties of the polymer blend groups. The lowest melt temperature that produced the highest flexural strength and flexural modulus was selected as the appropriate melt temperature for each group. Thus, according to our findings (Tables 1 and 2), the appropriate melt temperature of M100, M75, M50, M25, and M0 group were 270, 240, 230, 210, and 210 °C, respectively. The comparison between the groups of individuals from each blend ratio revealed that the flexural strength of polymer blends was comparable to that of neat PMMA and there was no difference in flexural modulus between each blend group and neat PLA.

**Table 1:** The PMMA/PLA blend ratios with different melt temperatures (T1–T4).

Group	Ratio (%mg)		Melt temperature (°C)			
	PMMA	PLA	T1	T2	T3	T4
M100	100	0	240	250	260	270
M75	75	25	240	250	260	265
M50	50	50	220	230	240	260
M25	25	75	210	220	230	255
M0	0	100	200	210	220	250

**Table 2:** Mean (SD) of flexural properties (MPa) of polymer blend groups.

		T1	T2	T3	T4
M100	Flexural strength	69.86^b^ (3.97)	74.12^b^ (1.82)	77.35^a,b^ (1.45)	77.93^a^ (3.90)
	Flexural modulus	2,948.00^b^ (235.51)	2,939.10^b^ (119.88)	3,073.63^b^ (105.92)	3,339.80^a^ (195.46)
	Mode of failure	F	F	F	F
M75	Flexural strength	77.98^a^ (2.18)	60.22^b^ (5.30)	63.42^b^ (3.00)	62.75^b^ (5.83)
	Flexural modulus	3,459.37^a^ (132.02)	3,310.44^a^ (197.96)	3,334.43^a^ (114.33)	3,274.63^a^ (361.84)
	Mode of failure	F	F	F	F
M50	Flexural strength	72.25^b^ (1.06)	77.25^a^ (3.85)	75.02^a,b^ (3.36)	36.75^c^ (5.04)
	Flexural modulus	3,368.99^b^ (72.77)	3,607.03^a^ (152.54)	3,602.66^a^ (69.49)	3,005.47^c^ (172.01)
	Mode of failure	F	F	F	F
M25	Flexural strength	76.22^a^ (0.93)	62.43^b^ (7.36)	47.92^c^ (4.52)	33.25^d^ (4.41)
	Flexural modulus	3,651.68^a^ (77.48)	3,584.55^a^ (119.01)	3,375.83^b^ (213.33)	3,086.25^c^ (163.67)
	Mode of failure	F	F	F	F
M0	Flexural strength	72.01^b^ (0.84)	73.21^a,b^ (1.25)	74.23^a^ (0.76)	35.41^c^ (2.69)
	Flexural modulus	3,583.38^a^ (88.50)	3,570.26^a^ (134.33)	3,663.87^a^ (67.85)	3,190.97^b^ (234.68)
	Mode of failure	T	T	T	F

*The same superscript letter means there is no significant difference between groups in each row. F means fracture and T means tear in mode of failure.

### Miscibility of polymer blend

**Figure 1:**
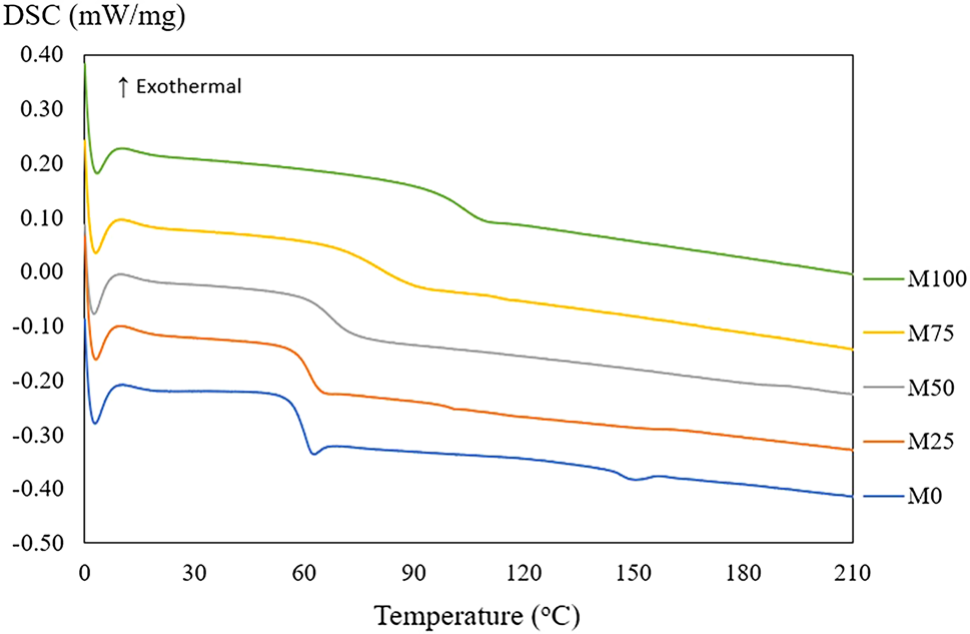
The DSC thermogram of M100, M75, M50, M25, and M0 groups.

The T_g_ of polymer beads from each group was investigated by DSC and displayed in the single value across all groups. M100 showed the highest T_g_ value followed by M75, M50, M25, and M0 (102.4, 80.8, 68.4, 61.8, and 59.6 °C, respectively). The T_g_ of polymer blends (M25, M50 and M75) were observed between neat polymers (M0 and M100) as showed in Fig. 1. Thus, the PMMA/PLA blend was found to be a miscible blend in all compositions in this study.

**Figure 2:**
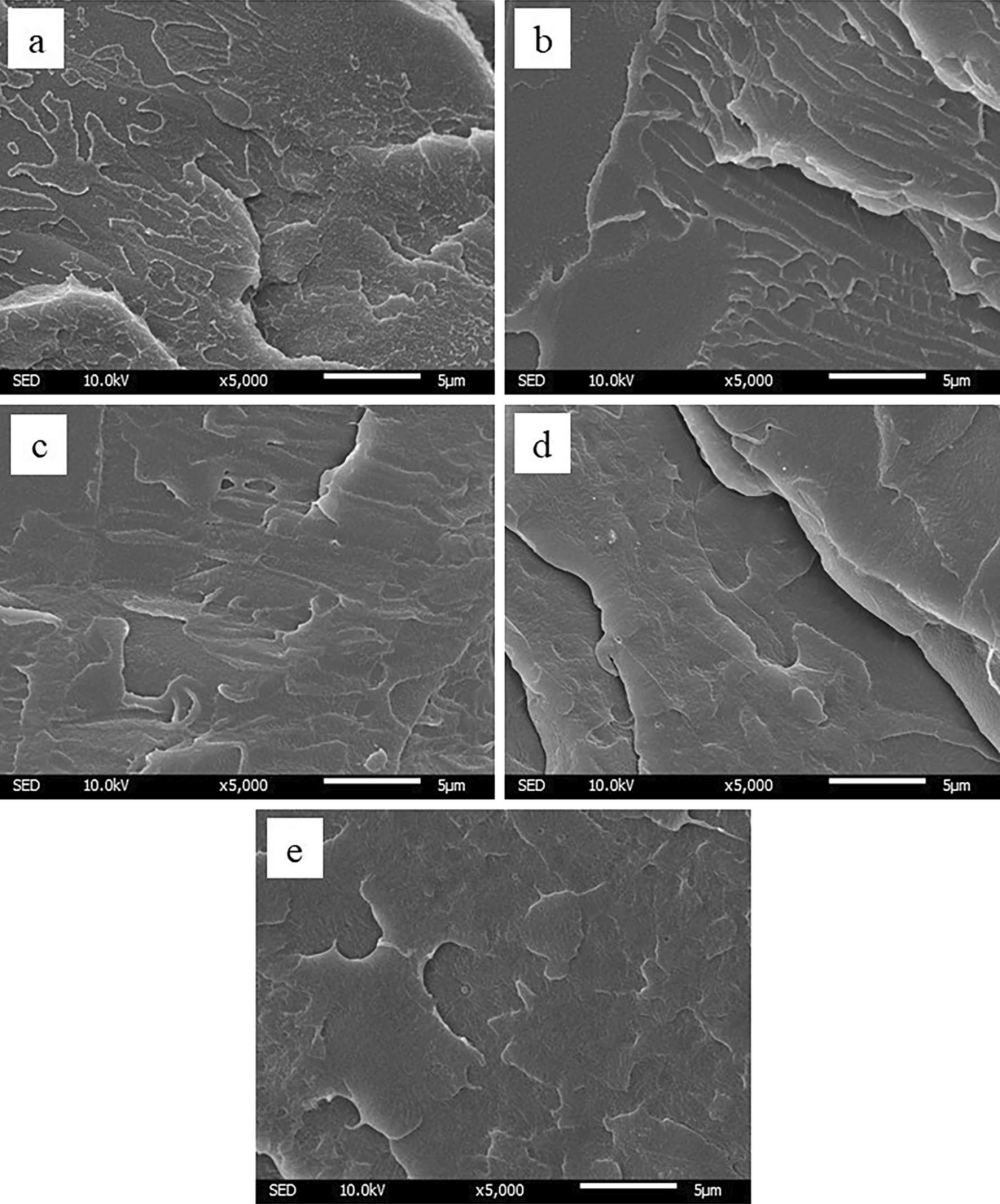
The SEM topography of cryo-fracture surface of polymer beads (5,000 × magnification) M100 (**a**), M75 (**b**), M50 (**c**), M25 (**d**), and M0 (**e**).

The miscibility of polymer blends observed by SEM (Fig. 2) showed that the fracture surfaces of all polymer beads displayed the homogeneity surface without phase separation. This implies that all polymer blend groups have good miscibility.

### Residual monomer

**Figure 3:**
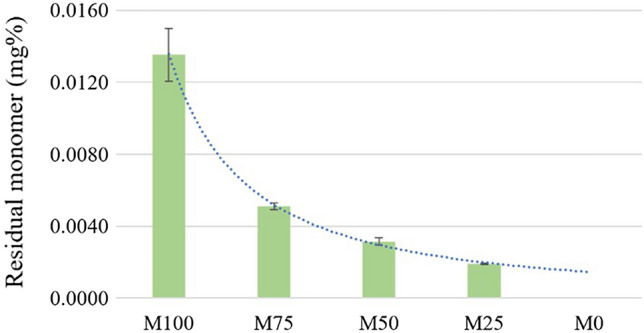
Mean and SD of residual monomer from each group (M0 − M100).

The standard calibration curve was drawn from standard solution. The equation for residual MMA determination was obtained from correlation coefficient (R^2^) equal to 1.00. Since group M0 (neat PLA) did not contain any PMMA, the result was reported as "N/A". The results demonstrated that the amount of residual monomer of all groups was significantly different from one another (Fig. 3). M100 group showed the highest released residual monomer while M25 group showed the lowest one.

### Water sorption and water solubility

**Figure 4:**
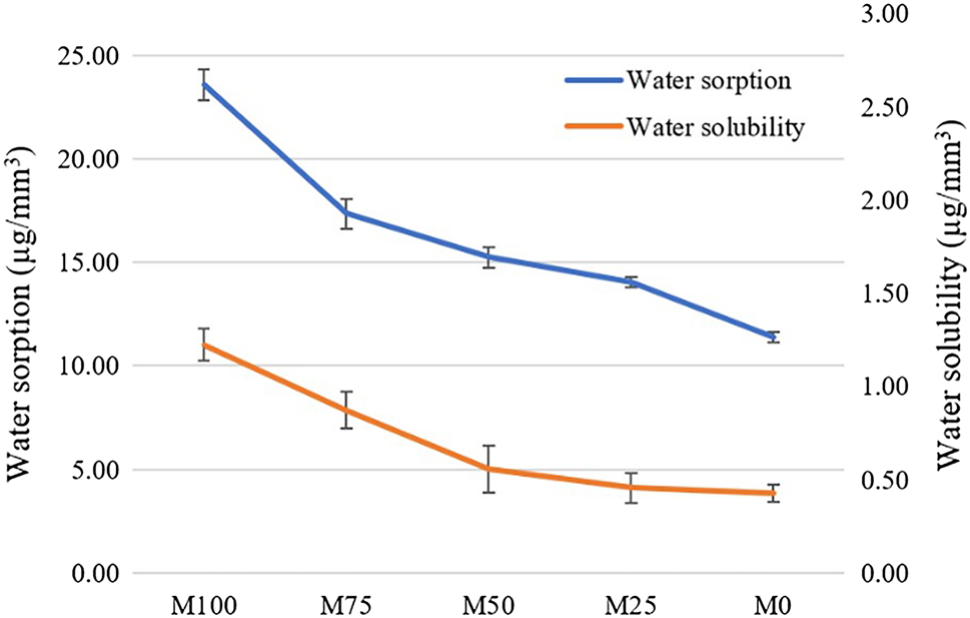
Water sorption and water solubility of each group (M0 − M100).

From the graph in Fig. 4, M100 showed the highest water sorption and water solubility while M0 showed the lowest water sorption and water solubility (*p* < 0.05). The water sorption and water solubility of the polymer blends were decreased when the amount of PLA reduced, and they are also different to each other (*p* < 0.05).

### Degradation

**Figure 5:**
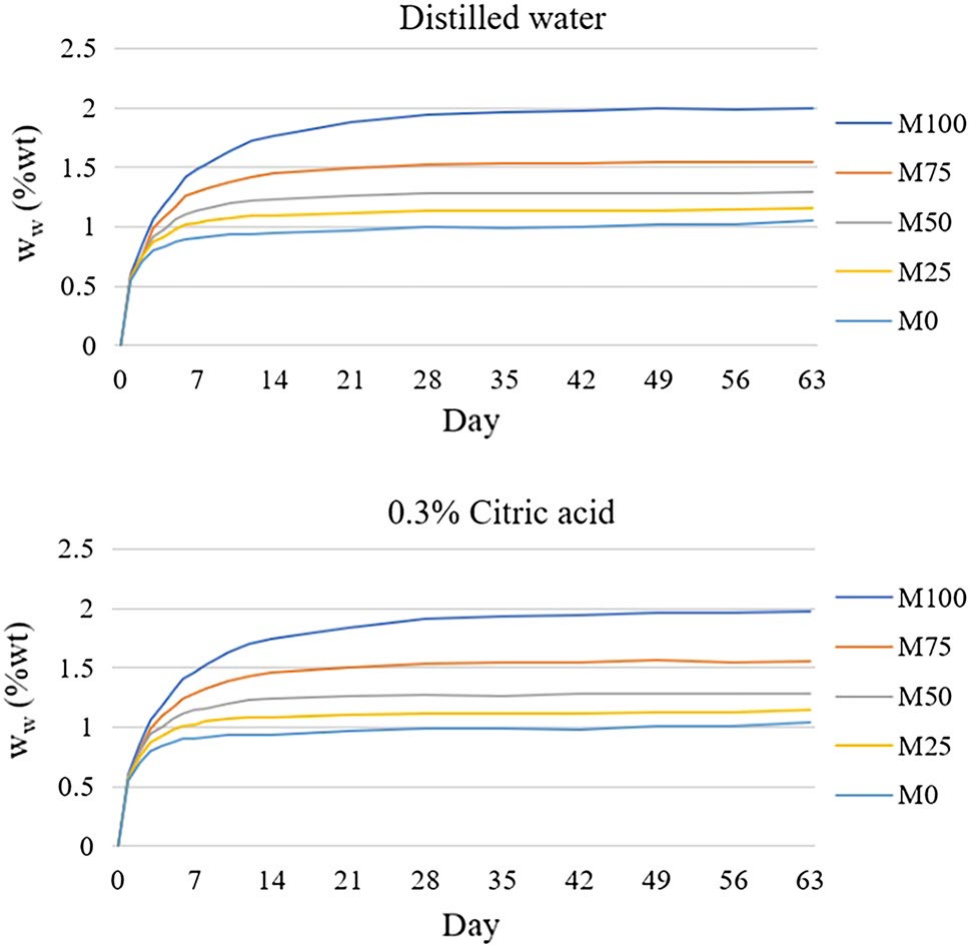
Graphs of weight change (w_w_) after immersion from day 0 till day 63 in distilled water and 0.3% citric acid solutions.

The weight changes of different polymer blend ratios after immersion in distilled water and 0.3% citric acid from day 0 till day 63 were displayed in Fig. 5.

The weight of the specimens significantly increased over the first week after immersion, regardless of solution. Then, the weight started to rise steadily until it reached a plateau on day 35. The percentage of weight change of specimen after immersion in solutions for 63 days (w_w_) was analyzed. Two-way ANOVA was performed to analyze the effect of ‘blend ration’ and ‘types of solution’. The results showed that blend ration was significantly affected after immersion for 63 days (*p* < 0.05). However, the type of solution showed no significant difference (*p* > 0.05). The M100 showed the highest weight of wet polymer (w_w_), followed by M75, M50, M25, and M0. The interaction between two factors was not observed (*p* > 0.05). Thus, one-way ANOVA and Tukey HSD were used to analyze the difference between groups. The results showed in Table 3.

**Table 3:** Mean (SD) of weight of wet polymer (w_w_, %) of each group (M0 − M100).

	M100	M75	M50	M25	M0
Distilled water	1.995^a^ (0.010)	1.542^b^ (0.009)	1.291^c^ (0.017)	1.152^d^ (0.013)	1.048^e^ (0.014)
0.3% Citric acid	1.970^a^ (0.016)	1.556^b^ (0.017)	1.286^c^ (0.016)	1.143^d^ (0.017)	1.042^e^ (0.010)

*The same superscript letter means no significant difference.

**Figure 6:**
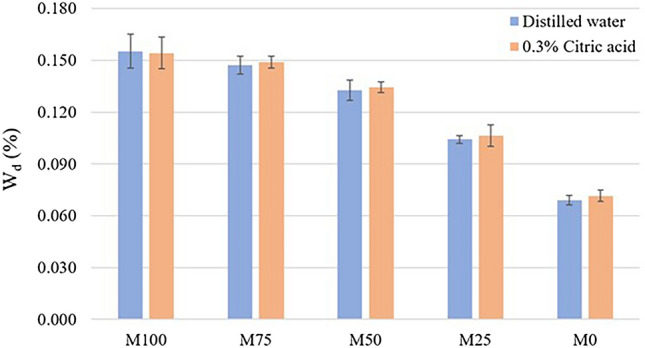
Mean and SD of dried weight (w_d_, %) in each group.

Analysis of the percentage of weight change of dried specimen after immersion in solutions (w_d_) exhibited normal distribution for all groups. Then, two-way ANOVA was performed. The result showed that solution did not affect the w_d_ (*p* > 0.05), but the blend ration had an effect on the dried weight (*p* < 0.05). The interaction between two factors was not found (*p* > 0.05). By analyzing with one-way ANOVA and Tukey HSD, the results showed that even though the weight loss of M100 and M75 was not significantly different, their weight loss was significantly higher than the others (*p* < 0.05). While the M0 showed the lowest weight loss (*p* < 0.05) shown in Fig. 6.

### Color stability

**Figure 7:**
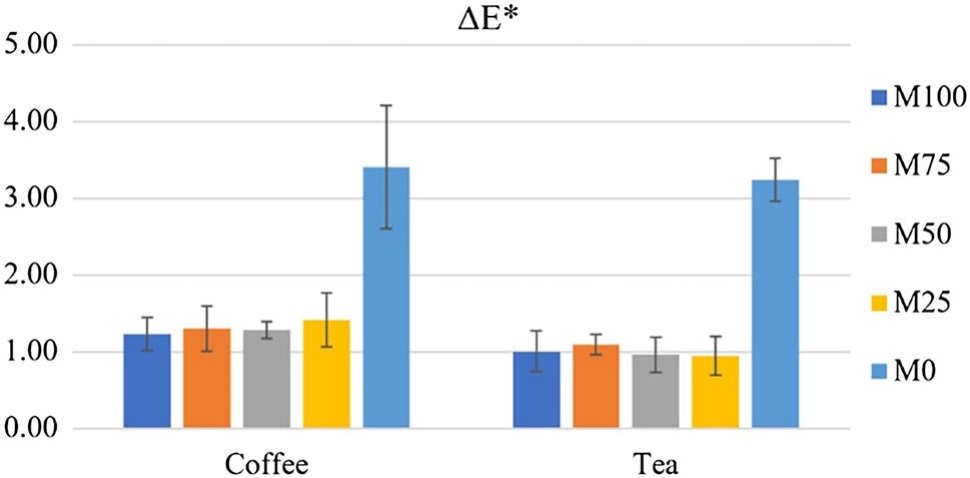
Mean and SD of color change (∆E*) of each group.

The results showed that the color change of M0 was much greater than that of other groups in both solutions (*p* < 0.05). However, there were no significant difference in color change between M100, M75, M50, and M25 (*p* > 0.05) (Fig. 7). Two-way ANOVA showed significantly difference in both ‘blend ratio’ and ‘solutions’ (*p* < 0.05) but no interaction between these factors (*p* > 0.05). However, coffee caused more significantly change in color than tea. Thus, the data was separately analyzed depending on solutions by one-way ANOVA with Tukey HSD.

### Biocompatibility

**Figure 8:**
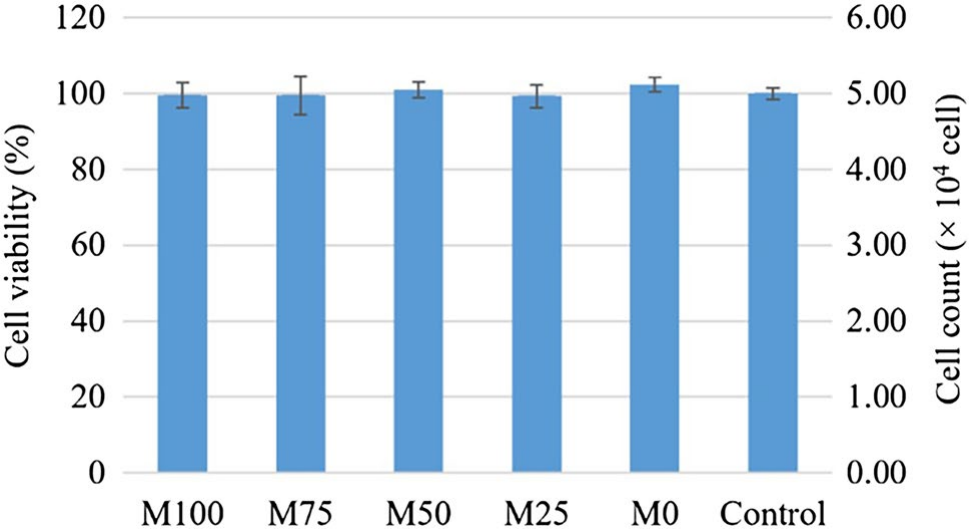
Cell viability (%) and amount of cell count (× 10^4^ cell) of each group.

The cell viability (%) of each group was calculated and compared to the control group. The results demonstrated that the elution in every test group was not toxic (% cell viability > 70), according to ISO 10993^19^. The cell count of each group was calculated by the standard equation [y = 0.028x + 0.0288]. The R^2^ was reported at approximately 0.96 which showed a strong correlation between OD and cell count. Then, the cell amount was compared between groups to define the statistical difference. The data was analyzed by one-way ANOVA and revealed no significant difference between groups (*p* > 0.05) Fig. 8.

## Discussion

The properties of the polymer blend are dependent on the miscibility of the blends. The miscible blend could either obtain the benefits of each polymer or improve the properties of polymer blends, while the immiscible blend can cause the poor properties^20^. Thus, the miscibility of the blend is one of the important factors. The miscibility could be investigated by many methods such as scanning electron microscope (SEM), Fourier Transform Infrared Spectroscopy (FTIR), Differential Scanning Calorimetry (DSC), X-ray Diffraction (XRD). The DSC and SEM were methods used in this study to investigate miscibility^21^.

The DSC showed single T_g_ value in all PMMA/PLA blend ratio. The T_g_ of PMMA/PLA blends were within the T_g_ range between neat PLA and PMMA. The T_g_ of polymer blends increased when the PMMA ratio increased. This is also found in the widening of T_g_. The endothermic peak of melting was only observed in M0 (neat PLA) at approximately 150 °C. This could be implied that neat PLA had crystalline phase of the structure. The endothermic peak in polymer blend groups could not be detected. This could be that the polymer chain arrangement in PLA was disturbed by amorphous PMMA leading to no PLA crystalline occurring. This phenomenon agreed with the previous study by Zhang et al., who observed the endothermic peak of melting temperature of PMMA/PLA blend when the composition of PLA was more than 90%^22^. Gonzalez-Garzon et al. also found that the crystallization of PLA in the polymer blend was restricted when the composition of PMMA was more than 30%^14^.

No evidence of phase separation was found in the fracture surface under SEM investigation, which also indicated homogeneity in all polymer blend ratios. This suggested that all PMMA/PLA blends used in this study were miscible blends. The miscibility of the polymer blend could be affected by other factors such as blending method. The polymer could be blended by either melting or mixing the polymer beads with screw extruder, called “melt-blending”. Another method is to dissolve the polymer beads with solvent, called “solution casting”^16^. Samuel et al., found that PLLA/PMMA blend with melt-blending followed by injection molding was miscible in all composition by shear force during blending by twin-screw. This could support the result of miscibility this study^16^.

To prepare the specimens, the injection molding technique using polymer beads was chosen. The suitable melting temperature and time in order to sufficiently melt and to be injected into the mold were selected. If the temperature was too low, the melted polymer might not fill the mold and caused defects of the specimen. In contrast, the high temperature could cause thermal degradation which lead to color changing, pore formation or decrease the mechanical properties^23,24^. Our study found that increasing the melting temperature could lead to a decrease in the flexural properties. The common failure of M0 was ‘Tear’ which was different from other groups. This failure might be caused from the testing temperature being close to the T_g_ of the PLA. This could make polymer more rubber-like and ductile^25^. But when M0 was melted at 250 °C, the failure mode of all test specimen was ‘Fracture’ and occurred at lower flexural properties than the others. This might be resulted from the thermal degradation of the polymers when fabricating at high temperature causing polymer more brittle^26^.

The flexural properties indicate the ability of material to resist fracture from the masticatory forces. In this study, the flexural strength of PMMA was higher than PLA but not different from the other polymer blends. In contrast to flexural strength, PLA showed higher flexural modulus than PMMA but was not different from the other polymer blends. Thus, mixing PMMA to PLA could gain the benefit of both polymers. The PMMA/PLA blend showed both comparable flexural strength to PMMA and comparable flexural modulus to PLA.

The previous studies showed that the 3D-printed PLA by FDM has lower flexural strength than PMMA^13,27^. Additionally, the flexural strength of the milled PLA was reported to be lower^28^ than that of milled PMMA was reported by the other studies^29,30^. This could be caused from the milled PLA block prepared by printing method, which printing orientation would affect the strength of material^31^. Unlike, the milled block or disc fabricated by other techniques instead of 3D printing could provide the optimum properties of the specimens^32,33^. Thus, the polymer beads fabricated in this study could be used as high strength raw materials.

Residual MMA monomer in polymerized acrylic resin could affect the mechanical and physical properties of denture base. It has shown that residual monomer acts as a plasticizer which can reduce flexural strength, increase water sorption and solubility, decrease color stability, and be toxic to human cells^2,34,35^. The conventional heat-polymerized PMMA showed residual monomer about 1.16–3.00 mg%^36,37^. When comparing to M100 (neat PMMA) the residual monomer was much lower than those studies. This may be due to the different types of raw material between polymer beads and powder-liquid polymer. The thermoplastic polymer bead used in this study was nearly completely polymerized. In contrast to the fabrication by melting and injecting thermoplastic polymer bead into specimen mold, fabrication by conventional heat polymerized PMMA using powder and liquid could produce more residual monomer after polymerization.

The amount of residual monomer observed in this study significantly decreased when the PMMA was blended with PLA. The detected residual monomer dramatically decreased from M100 to M75, and then gradually decreased from M75 to M50 and M25 accordingly. The decreasing of residual monomer in PMMA/PLA blend was as expected due to the PMMA component being reduced when PLA was blended. The previous study reported that the residual monomer could be affected by heat and time^38^. The residual monomer was obviously decreased from neat PMMA beads (M100) and PMMA/PLA blends (M75, M50, and M25). This could result from heat during fabrication of the PMMA/PLA blends which caused the residual monomer to reduce during polymerization. As previously described, the blend process was operated at 210 °C, and fabrication was performed between 210–270 °C which were above the boiling point of MMA (about 101 °C). This may cause either monomer evaporation or continuing polymerization. Even though lactic acid is used as component of solution to produce artificial caries in vitro, the residual lactic acid monomer was not investigated in this study^39^. The reason was that the concentration of lactic acid must be high enough to decrease the pH to critical value and the previous study reported that the leaching lactic acid from PLA was very low (about 0.24 µg/cm^2^ in 30 min)^40^. Thus, our finding could imply that blending the PLA to PMMA could significantly reduce the residual MMA monomer.

To investigate the water sorption and water solubility, it showed that M100 had the highest values while M0 had the lowest ones. The water sorption and water solubility decreased when the ratio of PLA was increased as indicated in Fig. 4. Salih et al. reported the water sorption and water solubility of PMMA denture base at 23.2 and 1.8 µg/mm^3^, respectively, which were closely to M100 in this study^41^. When considering the polymer structure of PMMA, the functional groups of PMMA (-COCH_3_) can make PMMA more hydrophilic due to being a polar group. PLA is strongly hydrophobic polymer even the ester group exists in backbone chain, but the side chain is methyl group^42,43^. High water sorption and solubility leads to a decrease in mechanical properties, and color stability of denture base materials. The water sorption into PLA networks can cause hydrolysis of the polymer backbone at ester site^12^. Thus, these properties of polymer should be as low as possible to maintain the properties of materials.

The mean weights of wet polymer (w_w_) and weight after degradation (w_d_) were not statistically different, regardless of solution. Thus, immersion in 0.3% citric acid, polymer blended was affected by water uptake and released the soluble substances as similar as in distilled water. However, the blend ration could have an influence on both weights of wet polymer and weight after degradation. This might be explained by the result of water sorption and water solubility which M0 exhibited the lowest value and M100 showing the highest weight change.

Color is another important property of materials. When exposed to staining substances found in foods and beverages, materials may lose their original color. The color change could be observed by visual perception or spectrophotometer. By using spectrophotometer, the color changed (∆E*) is calculated from different of L*, a*, and b*. The human eye could detect the difference in color when ∆E* is above 1.0^44^. However, Ruyter et al. reported that ∆E* above 3.3 was unacceptable in dentistry^45^. The coffee and tea were selected as representative of daily consumed beverages due to the staining ability of these two beverages was higher than other beverages^46,47^. In this study, color change of M0 group was the highest while all PMMA/PLA blend ratios showed no significant difference in color stability, compared to PMMA. Color stability of the denture base materials depends on many factors including surface roughness, T_g_, UV resistance, chemical composition, or water sorption^48,49^. According to the lower water sorption, water solubility and residual monomer of PLA than those of PMMA, the thermal characteristics and chemical resistance of material could be results in lower color stability of PLA.

Color change of materials could occur by staining at the surface or penetration of colorants into materials. As previously described, the T_g_ of PLA was about 55 °C which is closely to the testing temperature (37 °C), while PMMA/PLA blend showed higher T_g_ than neat PLA. When the environmental temperature is close to T_g_, the material appears to be softened. Consequently, the solutions can easily penetrate the bulk materials. Thus, PLA appears to be degraded by bulk erosion as a result of the inner core deteriorating more quickly than the outer region^50,51^. This suggested that PLA could be permeated by the solutions more than PMMA. As the present result, the colorants were able to enter the materials but were not easily removed by mechanical methods such as rinsing, scrubbing, or grinding.

The in vitro cytotoxicity tests are used as screening tests for biocompatibility. To investigate the cytotoxicity of dental materials, the result of MTT test showed no toxicity in all groups and no difference from each other. It has been well accepted that the cytotoxicity of the denture base materials was correlated with the amount of residual monomer. A previous study showed that PLA is biocompatible because of its degradation property. PLA can degrade into lactic acid or carbon dioxide (CO_2_) and water. These degradation products could be metabolized intracellularly and excreted through kidney filtration in urine and breath^52^. This study confirms that the neat polymers and the polymer blends were not toxic. This may be due to the relatively low level of residual monomer. Thus, PMMA, PLA, and PMMA/PLA blends in this study could be an alternative material for dental application with good biocompatibility.

From this study, the PMMA/PLA blend polymer beads could be used as raw materials for manufacturing processes such as injection molding and digital fabrication in dentistry. PMMA/PLA beads could be either formed into disc shape or ingot for CNC fabrication. Furthermore, PMMA/PLA beads could be prepared into filament, powder-liquid, and liquid resin for polymer 3D printing. However, this blended biomaterial may need some further modification to meet the requirements of each application such as color, characteristic, translucency. Therefore, a variety of dental applications can be further developed. Additionally, the thermoplastic materials could be recycled to reduce waste from manufacturing, and the biodegradable properties of PLA still existed after blended to PMMA. Thus, these polymer blends are more environment friendly than conventional PMMA and could be introduced to more dental applications in the future to promote sustainability.

Due to the growth in digital fabrication in manufacturing process and environmental concern, the properties of materials, especially the strength of material, should be considered. The benefits of PLA are biocompatibility, biodegradable, physical and mechanical properties close to PMMA. This PMMA/PLA blend could have potential applications in the dental field, including denture base materials, temporary restorations, or possibly as a component in dental implants or bone graft substitutes.

According to the result of PMMA/PLA blends (M25, M50 and M75) in this study, the M25 is not suitable for long-term temporary or permanent restoration due to the low glass transition temperature. Even though M75 had higher T_g_ than M50, the flexural properties, color stability and biocompatibility were not different from each other. The M50 showed less residual monomer, water sorption, water solubility, and degradation than M75. Furthermore, the melt temperature of M50 was less than that of M75 which can be benefit due to less energy required for fabrication. From our finding, M50 ratio could be a suitable PMMA/PLA blend ratio for long-term temporary and permanent restoration in dental applications. However, it is necessary to do further investigation on the different ratios between M50–M75 to optimize the properties of polymer blend in this range and find the most favorable ratio.

## Material and methods

### Polymer blend preparation

The PMMA polymer beads with average molecular weight (*M*_*w*_) of 11.0 × 10^4^ g/mol (Acrypet™ MD001, Diapolyacrylate Co. Ltd, Thailand), and PLA polymer beads with average molecular weight of 11.6 × 10^4^ g/mol (Ingeo™ Biopolymer 3052D, NatureWorks LLC, USA) were used in this study. To prepare PMMA/PLA polymer blend, the PMMA and PLA beads were melt-blended using twin-screw extruder (Collin T-20, Maitenbeth, Germany) in different ratios at 210 °C, 60 rpm for 10 min under dried nitrogen airflow. There were 5 different ratios of PMMA/PLA to be investigated (Table 1). The polymer blend beads of each group were loaded into stainless steel tube and fabricated into specific shapes for further investigations using dental injection unit (Myerson Flex Press, Myerson LLC, USA). The polymer beads were melted at melt temperature for 20 min, then the melted polymer was injected into the mold with the 8 bar of pressure and was held the pressure for 10 min.

### Flexural properties testing

To find the most favorable melt temperature for these thermoplastic polymer groups, the flexural properties of 5 groups of PMMA/PLA blend ratio were investigated in coordination with 4 different melt temperatures obtained from the preliminary study and were defined as T1, T2, T3, and T4 (Table 1). The lowest temperature at which a specimen could be manufactured without having any flaws, such as a weld line or an incomplete injection, was determined as the lowest melt temperature. The highest melt temperature was determined by the temperature at which the color of specimen did not change, signifying that the specimen had not undergone thermal degradation.

Ten bar-shape specimens of each group sized 10.0 × 64.0 × 3.3 (± 0.2) mm were fabricated and polished using silicon carbide paper up to P1200. All specimens were kept in 37 °C water in incubator for 48 ± 2 h prior to flexural properties testing. The specimens were tested using the universal testing machine Shimadzu EZ-S (Shimadzu, Kyoto, Japan) with load cell 500 N at crosshead speed 5 mm/min in 37 °C water. The maximum flexural strength and flexural modulus were recorded. The failure mode of each specimen was classified as either fracture (F) or tear (T).

### Blend miscibility testing

The miscibility was observed in 2 aspects including thermodynamic properties and topographic investigation. First, the thermodynamic properties of polymer beads were investigated by Differential Scanning Calorimetry (DSC). The polymer beads of each group were fragmented into 10 mg and analyze by DSC 204 F1 Phoenix (NETZSCH-Gerätebau GmbH, Selb, Germany). The samples were processed under nitrogen gas with heat rate at 10 °C/min from 0 − 220 °C to erase thermal history and cool down rate at 10 °C/min to 0 °C. The cooling and second heating scans were used to evaluate the material’s ability to crystallize and the glass transition temperatures. The melting temperature of each group was also determined.

The other miscibility aspect was investigated by Scanning electron microscope (SEM). The polymer bead of each group was immersed in liquid nitrogen and later broken into pieces. The fracture surfaces of each specimen group were gold-coated and observed under SEM with JSM-IT300 InTouchScope (Joel Ltd., Tokyo, Japan) at 5000 × magnification.

### Residual methyl methacrylate (MMA) monomer testing

Six specimens of each group were fabricated into disc shaped size 50 mm in diameter and 2 mm thickness. The residual monomer test was conducted in accordance with ISO 20795–1. To serve as the m_sample_ for calculation, the samples from each group were given a 650 mg weighting. The residual MMA monomer was analyzed by high-performance liquid chromatography (HPLC) system with 5 μm particle size, 250 mm length and 4.6 mm internal diameter column. The amount of residual MMA monomer (m_MMA_) was obtained from the standard calibration curve. Therefore, the residual monomer (%mg) was calculated from equation.

$$ {\text{Residual}}\;{\text{monomer}} = \left( {{\text{m}}_{{{\text{MMA}}}} {\text{/m}}_{{{\text{sample}}}} } \right) \times {1}00 $$

### Water sorption and water solubility testing

Five specimens of each group were fabricated as 50 mm diameter, 0.5 mm thick disc and polished using the same previous procedure. The specimens were kept in desiccator containing freshly dried silica gel in 37 °C incubator and frequently weighted until the mass remained constant, at which point “m_1_” was recorded. The volume of specimen (V) was calculated using the mean of three measurements of diameter and the mean of five measurements of thickness. The test method complied with ISO 20795–1, so the specimens were immersed in 37 °C water for 168 h. The specimens were dry towel cleaned and weighted as “m_2_”. The specimens were reconditioned to constant mass in the desiccator and recorded as “m_3_”. The water sorption (w_sp_) and water solubility (w_sl_) were calculated by following equation:

$$ {\text{Water}}\;{\text{sorption}}\;\left( {{\text{w}}_{{{\text{sp}}}} ,\;\upmu {\text{g/mm}}^{{3}} } \right) = \left( {{\text{m}}_{{2}} {-}{\text{m}}_{{3}} } \right){\text{/V}} $$

$$ {\text{Water}}\;{\text{solubility}}\;\left( {{\text{w}}_{{{\text{sl}}}} ,\;\upmu {\text{g/mm}}^{{3}} } \right) = \left( {{\text{m}}_{{1}} {-}{\text{ m}}_{{3}} } \right){\text{/V}} $$

### Degradation test

To investigate the degradation of polymer blends, six disc-shaped specimens of each group sized 50 mm diameter and 2.0 mm thick were prepared following the same procedure. The specimens were also kept in desiccator and the specimens were weighed until their mass remained constant, at which point "d_0_" was recorded. The specimens were randomly divided into 2 groups and immersed in sealed container with either distilled water or 0.3% citric acid at 37 °C incubator for 63 days. The solutions were changed every week and the specimens were rinsed with distilled water before immersing in the fresh solutions. The specimens were weighted every day from day 1 till day 8. Then, the specimens were weighted at day 10, 12, 14, 21, 28, 35, 42, 49, 56, and 63. The specimens were taken out of the solution, wiped with a dry clean cloth, waved in the air for 15 ± 1 s and then weighed in 60 ± 10 s. The weight of day 63 was recorded as “d_63_”. The weight of wet polymer (w_w_) was calculated as the following equation.

$$ {\text{Weight}}\;{\text{of}}\;{\text{wet}}\;{\text{polymer}}\;({\text{w}}_{{\text{w}}} ,\;\% ) = \left( {{\text{d}}_{{{63}}} {-}{\text{d}}_{0} } \right){\text{/d}}_{0} \times {1}00 $$

The samples were restored to dried constant mass in the desiccator after day 63. The dried weight was recorded as “d_f_”. The final weight change (w_d_) was calculated as the following equation.

$$ {\text{Weight}}\;{\text{change}}\;\left( {{\text{w}}_{{\text{d}}} ,\;\% } \right) = ({\text{d}}_{0} {-}{\text{d}}_{{\text{f}}} ){\text{/d}}_{0} \times {1}00 $$

### Color stability

To observe the color stability, twelve square-shaped specimen size 10.0 × 10.0 mm with 2.0 mm thick were fabricated following the same as previous procedure and randomly divided into 2 groups of two different solutions (coffee and tea). The baseline value of the color of specimens was evaluated by UltraScan Pro Spectrophotometer (Hunter Association Laboratory Inc., Virginia, USA) using CIE Lab system (International commission on Illumination), port 7 mm (Small area view: SAV), and reflectance specular included (RSIN). Three measurements of each specimen were taken, and the average value was recorded. The specimens were then immersed in either coffee or tea for 7 days at 37 °C in incubator. The specimen was rinsed with distilled water for 30 s, dried with towel and waved in the air for 15 s. The color measurements of specimens were repeated and compared to baseline values of each specimen. The difference of color (ΔE) was calculated using the equation.

$$ {\text{Difference}}\;{\text{of}}\;{\text{color}}\;\left( {\Delta {\text{E}}^*} \right) = \left( {\Delta {\text{L}}^{*{2}} + \, \Delta {\text{a}}^{*{2}} + \, \Delta {\text{b}}^{*{2}} } \right)^{{{1}/{2}}} $$

### Biocompatibility

The MTT cytotoxicity test following ISO 10993–5:2009 was selected. The human fibroblast cells were harvested from healthy patient’s teeth who received surgical removal of third molar at Department of Surgery, Faculty of Dentistry, Chulalongkorn University. The inform consent was also obtained from the participant and/or their legal guardians. All methods were conducted in accordance with the relevant guidelines and regulations of the Institutional Review Board. The protocol was approved by Human Research Ethics Committee, Faculty of Dentistry, Chulalongkorn University (HREC-DCU 2022–013). The extracted teeth were immersed in normal saline solution and immediately cultured in Dulbecco’s Modified Eagle’s Medium (DMEM) with 10% Fetal bovine serum (FBS), 1% L-Glutamine, and 1% Antibiotic & Antimycotic (100 U/mL penicillin, and 100 µg/mL streptomycin). The human fibroblast cells were cultured from dental pulp, periodontal ligament, and gingival tissue. Cultures were performed at 37 °C in a humidified atmosphere of 5% CO_2_ and 95% air.

The sub-cultured cells were seeded 4 × 10^4^ cells per well in 24-well plates (5 types of polymer bead, and 1 positive control group). The cytotoxicity was evaluated with indirect method MTT assay. The experiments were performed in triplicate (n = 3). The elution of specimens was prepared by immersion 0.5 g of polymer beads in 2.5 mL of DMEM without serum (N), and 10% FBS-DMEM (S) for 24 ± 2 h at 37 ± 1 °C. The conditioned mediums of each group were centrifuged at 3,000 rpm for 2 min and transferred to 15 mL centrifugal tube through 0.2 µm filter. Then 0.5 mL of conditioned media was treated to the seed cell for 24 ± 2 h and left untreated cell as positive control group. After that, the conditioned media was pipetted out. 0.5 mL of MTT solution (1.0 mg/mL in phosphate-buffered saline) was added to each well and incubated for 30 min and pipetted out. Then, 0.5 mL of dimethyl sulfoxide solution (DMSO) was added. The absorbance (OD) of each mixture was read with UV spectrophotometer at 570 nm. The percentage of cell viability was calculated by the equation.

$$ {\text{Cell}}\;{\text{viability}}\left( \% \right) = \left( {{\text{OD}}_{{{\text{test}}}} {\text{/OD}}_{{{\text{control}}}} } \right) \times {1}00 $$

The cell count of each well was calculated from a standard calibration curve that plotted from OD and number of cells.

### Statistical analysis

The data obtained from the flexural properties, residual monomer, water sorption, water solubility, degradation, color stability, and biocompatibility were analyzed by one-way ANOVA with Tukey HSD or Tamhane T2 test.

## Conclusions

When comparing the properties of neat PMMA, neat PLA, and PMMA/PLA blends, it can be concluded that PMMA/PLA blend with ratio of 50:50 can be suitable for being a sustainable biomaterial for dental applications. We can also summarize as the followings:

1. All groups of polymer blend (PMMA/PLA) were miscible regardless of ratio.
2. The T_g_ of PMMA/PLA blends was higher than the highest intraoral temperature during days.
3. The flexural strength of PMMA/PLA blend was comparable to PMMA with higher flexural modulus.
4. The PMMA/PLA blends have color stability comparable to PMMA but exhibited lower degradation than PMMA.
5. Water sorption and water solubility of PMMA/PLA blends were significantly lower than PMMA.
6. The residual monomer and biocompatibility of PMMA/PLA blends did not differ from one another.

## Data Availability

The supplement data is available upon request and should be addressed to S.V.
